# High-dose chemotherapy with stem cell rescue to treat stage III homologous deficient breast cancer: factors influencing clinical implementation

**DOI:** 10.1186/s12885-022-10412-x

**Published:** 2023-01-07

**Authors:** Joost G. E. Verbeek, Vincent M. T. de Jong, Hanna M. Wijnja, Agnes Jager, Sabine C. Linn, Valesca P. Retèl, Wim H. van Harten

**Affiliations:** 1 Division of Psychosocial Research and Epidemiology, The Netherlands Cancer Institute, P.O. Box 90203, 1006 BE Amsterdam, The Netherlands; 2 Department of Health Technology and Services Research, University of Twente, Enschede, The Netherlands; 3 Department of Molecular Pathology, Antoni Van Leeuwenhoek Hospital - Netherlands Cancer Institute, Amsterdam, The Netherlands; 4 Department of Medical Oncology, Erasmus MC Cancer Institute, Rotterdam, The Netherlands; 5 Department of Medical Oncology, Antoni Van Leeuwenhoek Hospital - Netherlands Cancer Institute, Amsterdam, The Netherlands; 6 Department of Pathology, Utrecht University Medical Centre, Utrecht, The Netherlands

**Keywords:** Breast Neoplasms, Homologous Recombination, Drug Therapy, Translational Research, Qualitative Research, Implementation Science

## Abstract

**Background:**

High-dose chemotherapy with autologous stem cell rescue (HDCT) is a promising treatment for patients with stage III, HER2-negative, homologous recombination deficient (HRD) breast cancer. Clinical effectiveness and cost-effectiveness are currently under investigation in an international multicenter randomized controlled trial. To increase the chance of successful introduction of HDCT into daily clinical practice, we aimed to identify relevant factors for smooth implementation using an early comprehensive assessment framework.

**Methods:**

This is a qualitative, multi-stakeholder, exploratory research using semi-structured interviews guided by the Constructive Technology Assessment model, which evaluates the quality of a novel health technology by clinical, economic, patient-related, and organizational factors. Stakeholders were recruited by purposeful stratified sampling and interviewed until sufficient content saturation was reached. Two researchers independently created themes, categories, and subcategories by following inductive coding steps, these were verified by a third researcher.

**Results:**

We interviewed 28 stakeholders between June 2019 and April 2021. In total, five overarching themes and seventeen categories were identified. Important findings for optimal implementation included the structural identification and referral of all eligible patients, early integration of supportive care, multidisciplinary collaboration between- and within hospitals, (de)centralization of treatment aspects, the provision of information for patients and healthcare professionals, and compliance to new regulation for the BRCA1-like test.

**Conclusions:**

In anticipation of a positive reimbursement decision, we recommend to take the highlighted implementation factors into consideration. This might expedite and guide high-quality equitable access to HDCT for patients with stage III, HER2-negative, HRD breast cancer in the Netherlands.

**Supplementary Information:**

The online version contains supplementary material available at 10.1186/s12885-022-10412-x.

## Introduction

Prognosis of stage III triple-negative breast cancer patients is still poor [1, 2, 3]. In the 1980s-1990s, phase-2 studies supported the concept that patients with metastasized or high-risk primary breast cancer could significantly benefit from chemotherapy dose escalation utilizing autologous stem cell rescue [4, 5]. Due to high public pressure, insurance companies in the United States were legally mandated to reimburse high-dose chemotherapy with autologous stem cell support (HDCT) for stage III BC patients [6]. However, after a fraudulent trial and numerous negative phase-3 trials, HDCT for breast cancer was considered obsolete [7, 8].

Objectively, HDCT has only shown limited clinical benefit in high-risk breast cancer, at the expense of considerable toxicity during the first year after diagnosis, and long-term toxicity comparable to conventional chemotherapy [8, 9, 10]. For example, pre-planned analyses of Dutch and American randomized controlled trials showed that HDCT improved 5-year relapse-free survival up to 10% for breast cancer patients with 10 or more positive lymph nodes [11, 12]. Moreover, in the triple-negative subset, tumors without hormone receptor expression nor HER2 overexpression, HDCT led to a 15.4% absolute survival benefit at 20 years [9].

Strikingly, in an unplanned subgroup analysis of patients with homologous recombination deficient (HRD) tumors, *e.g.* germline *BRCA1* or *BRCA2* mutation associated or “BRCA-like tumors”, the survival benefit is much larger [13]. This fits the paradigm that DNA-damaging agents, *e.g.* alkylating chemotherapy and radiotherapy may have an substantial advantage in the treatment of HRD tumors [13, 14, 15]. More specifically, the 7-year recurrence-free survival improved from 30% with conventional chemotherapy to 78% with intensified alkylating chemotherapy for breast cancer patients with a HRD (hazard ratio 0.12 (95% CI 0.04–0.43)), while no benefit was found for non-HRD patients [14]. These findings were consistent within two other subsequent retrospective studies [15, 16], but needs to be confirmed in a randomized controlled trial to be labeled as ‘established medical science and medical practice’ by the Dutch healthcare institute [17].

Therefore, the ongoing SUBITO trial (NCT02810743) randomizes patients with stage III, HER2 negative, HRD tumors to the current Dutch standard of care plus a Poly (ADP-ribose) polymerase (PARP) inhibitor (olaparib) or to HDCT. This trial investigates multiple outcomes, such as prognosis, quality of life, effect of chemotherapy on cognition, and cost-effectiveness. The SUBITO trial is part of a Coverage with Evidence Development (CED) trajectory due to the promising results in the retrospective analyses. This means that HDCT is conditionally reimbursed, targeted data is collected, and a timely reimbursement decision will be made after the study [18].

Generally, Health Technology Assessment (HTA) is used to evaluate medical, economic, organizational, social and ethical aspects to help provide evidence for decision-making and develop guidance on reimbursement [19]. HTA methods are commonly performed in mature health technologies that have proven efficacy and safety, but may have little impact on the development of the health technology. One framework considered an “early HTA” is Constructive Technology Assessment (CTA) [20]. This early framework can especially be used as an evaluation tool within CED programs and may be used before and during the introduction of a technology [20, 21, 22], when choices are constantly being made about the function, form, and use of that technology [23]. The CTA framework suits the evaluation of HDCT since this treatment is novel in the area of solid tumors, which requires a dynamic framework to enable researchers, healthcare professionals, and decision-makers to react to changes to the health technology, and to the environment surrounding the technology [22].

To increase the chance of a successful introduction of HDCT into daily clinical practice, good preparation and planning of implementation activities are necessary [24]. Hence, our objective here is to identify implementation factors for HDCT using a comprehensive early assessment framework, to ultimately guide and expedite optimal implementation of HDCT in the Netherlands.

## Methods

### Study design and framework

This is a qualitative exploratory, multi-stakeholder research [25], guided by the CTA framework described by Douma et al. (2007) [22], which uses the following four themes to evaluate novel health technologies: clinical, economic, patient-related, and organizational [26, 27, 28]. Each theme is subdivided into multiple categories, of which a detailed overview with descriptions can be found in Appendix A.

### Semi-structured interviews

We constructed a guide for the semi-structured interviews allowing for discussion with the interviewee. The guide consisted of five Sects. 1) interviewees characteristics, 2) organizational aspects, 3) clinical aspects, 4) economic aspects, 5) and patient-related aspects. Additionally, in-depth questions in the area of expertise of the interviewee(s) were prepared to stimulate discussion and understanding. A Dutch and English version of the interview guide can be found in Appendix B.

The questions were prepared by means of literature, research team meetings, and information from sounding board meetings with stakeholders of the SUBITO trial as part of the CED program. The final question set was developed after feedback from two HTA experts (VR, WVH), a lay-person, and we pilot tested the question set on VdJ. All interviews lasted one hour at most and were conducted individually, except for four interviews with healthcare professionals, which were held in small groups (two or three per interview) to accommodate efficiency in time and schedules. Two researchers (JV & VdJ or VR) conducted the interviews, except for three interviews that were conducted by one researcher (JV).

All interviewees agreed to anonymized audio-recording and transcription. This study received ethics approval by means of the protocol of the SUBITO trial (NCT02810743), the Netherlands Cancer Institute, the Netherlands, Amsterdam.

### Study sample

All participants were recruited by purposeful stratified sampling [29, 30]. Therefore, all interviewees were knowledgeable about HDCT and we intentionally included different perspectives including healthcare professionals, patients, patient representatives, policy-makers, and researchers. The interviewees were identified through the SUBITO consortium except for patients, whom were contacted by their treating medical oncologists on our request. We interviewed stakeholders until sufficient saturation of responses regarding the content was reached, meaning no new implementation factors were mentioned in additional interviews [30, 31].

### Analysis

The interviews were transcribed and labelled using NVivo version 1.4.1. (QSR International Pty Ltd., Doncaster, Australia). For the content analysis two researchers (JV, HW) independently created themes, categories, and subcategories by following the inductive coding steps described by Thomas (2006) [31]. Discrepancies were discussed until inter-coder agreement was reached. Furthermore, a third researcher (VdJ) verified the creation, overlapping, and refinement of the themes, categories and subcategories. Last, the created labels were counted per stakeholder group.

To display the findings, we constructed two tables: 1) implementation factors mentioned in at least half of the interviews within one or more of the stakeholder groups, and 2) implementation factors mentioned in less than half of the interviews within one of the stakeholder groups, but deemed important through the following ‘importance criteria’: 2A) evident risk for successful implementation of the health technology, 2B) potential impact on patient or treatment, and 2C) its relevance for research and development.

## Results

### Characteristics of participants

We conducted in-depth semi-structured interviews with 28 stakeholders between June 2019 and April 2021. The interviewees were eight medical oncologists, two specialized oncology nurse practitioners, three research nurses, one hematologist, one hospital pharmacist, three patients, two patient representatives, three scientists, and five policy-makers. They had an average age of 47 years (range: 30–73) and all but one policy-maker noted to be knowledgeable about HDCT.

All healthcare providers were employed in the Netherlands by either a university hospital, a specialized oncology center, or a teaching hospital and had on average 10 years (range: 1–25) hands-on experience with the treatment.

### Overarching themes

**Fig. 1:**
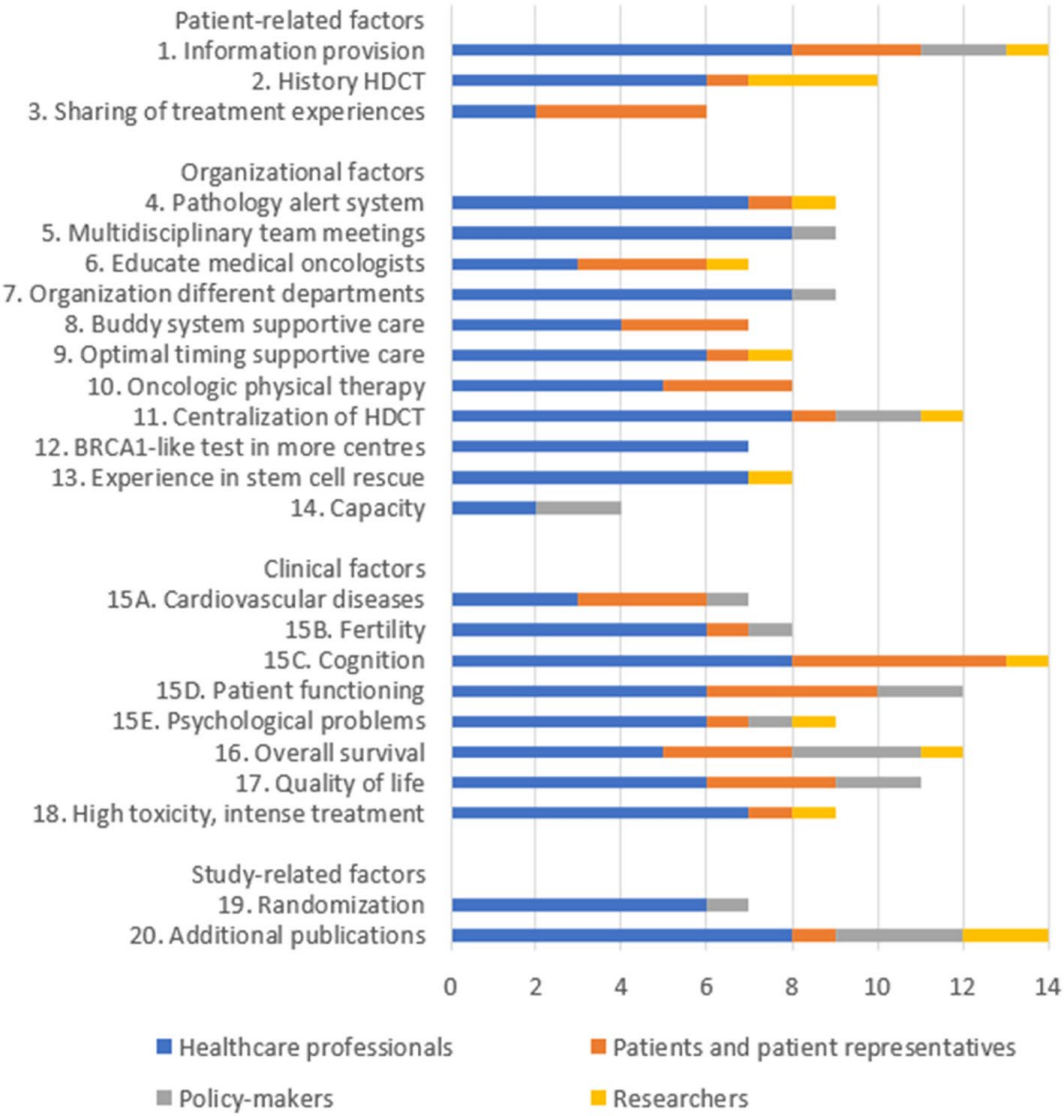
Most mentioned implementation factors by the four stakeholder groups. Cumulative representation

In total, we identified five overarching themes in the interviews, seventeen categories, and eighty-eight subcategories (Appendix C). Table 1 and Fig. 1 addresses all implementation factors mentioned by at least 50% of respondents within one stakeholder group, Table 2 summarizes less mentioned factors, but which passed the importance criteria.

**Table 1:** Identified implementation factors for the early application of high-dose chemotherapy with autologous stem cell rescue in the Netherlands as mentioned by at least half of the respondents within at least one stakeholder group

	**Themes and implementation factors**	**Categories**	**Healthcare professionals** **(*n* = 11**^**a**^**)**	**Patients and patientrep** **(*n* = 5)**	**Policy-makers** **(*n* = 4)**	**Researchers** **(*n* = 3)**	**Importance criteria**^**d**^
**No**	**Theme: Patient-related factors**		**N**^**b**^	**N**	**N**	**N**	**Value**
1	Clear information provision necessary for this complex treatment via leaflets, visual aids and/or websites	Provision of information	**8**^**c**^	**3**	**2**	1	B, C
2	A negative sentiment of high-dose chemotherapy due to the history of the treatment	Treatment perception	**6**	1	0	**3**	A, B
3	Sharing of treatment experiences between patients and treating medical oncologists	Treatment perception	2	**4**	0	0	B
**Theme: Organizational factors**							
4	The use of a pathology alert systems other alerts to create awareness of ongoing trials	Identification of patients	**7**	1	0	1	A, B, C
5	Multidisciplinary team meetings with (all) regional hospitals to increase inclusion rates	Identification of patients	**8**	0	1	0	B
6	Educate (referring) medical oncologists about the treatment, trial, eligibility criteria and prognoses	Referral of patients	3	**3**	0	1	B
7	Clear communication, responsibilities, and cooperation between and within departments (*i.e.*, medical oncology, haematology, radiology, surgery, nurses, quality managers & hospital pharmacy)	Organization of HDCT	**8**	0	1	0	A, B
8	One dedicated professional, and specialized “buddy system” in supportive care	Supportive care	4	**3**	0	0	B, C
9	Optimal timing, necessity and duration of supportive care for this treatment is unknown	Supportive care	**6**	1	0	1	B, C
10	Patients would benefit from oncologic physical therapy	Supportive care	5	**3**	0	0	B
11	Centralize HDCT for quality purposes (*i.e.* use of accreditation, guidelines, & quality managers)	Nationwide organization	**8**	1	**2**	1	A, B, C
12	BRCA1-like test can be performed in all centres if acquainted with MLPA	Nationwide organization	**7**	0	0	0	A, C
13	Experience on ASCT in the treating centre is required	Education	**7**	0	0	1	A, B, C
14	The specific capacity for ASCT like the amount of apheresis equipment, beds, and trained personnel are important	Capacity	2	0	**2**	0	A, B
**Theme: Clinical factors**							
15	Attention for short- and long-term effects:						
	-Effect of HDCT on cardiovascular diseases (*e.g.*, dyslipidemia, arrhythmia, high blood pressure)	Side-effects and adverse events	3	**3**	1	0	A, B, C
	-Effect of HDCT on fertility		**6**	1	1	0	
	-Effect of HDCT on cognition (*e.g.* concentration problems, chemobrain, etc.)		**8**	**5**	0	1	
	-Effect of HDCT on patient functioning (*e.g.* effect on work, relationships, etc.)		**6**	**4**	**2**	0	
	-Effect of HDCT on psychological problems (*e.g.* trauma, depression, anxiety etc.)		**6**	1	1	1	
16	Overall survival is most important for patients	Effectivity of the treatment	5	**3**	**3**	1	A, B, C
17	Quality of life after the treatment should also be taken into consideration	Effectivity of the treatment	**6**	**3**	**2**	0	A, B, C
18	A high toxicity, intense treatment is acceptable when prognosis significantly improves	Intensity of the treatment	**7**	1	0	1	A, B, C
**Theme: Study-related factors**							
19	Randomization might withhold patients from participating with the SUBITO study	SUBITO study	**6**	0	1	0	B, C
20	Additional publications on high-dose chemotherapy help with treatment acceptance among healthcare providers	SUBITO study	**8**	1	**3**	**2**	A, C

*HDCT* High-Dose Chemotherapy with Autologous Stem Cell Rescue, *mCTC* Mini Cyclophosphamide, Thiotepa, Carboplatin, *MLPA* Multiplex Ligation-dependent Probe Amplification, *PALGA* Pathologisch-Anatomisch Landelijk Geautomatiseerd Archief

^a^In total we interviewed sixteen healthcare professionals in eleven separate interviews

^b^N = Number of interviews in which this implementation factor has been mentioned within a stakeholder group

^c^All numbers in bold are factors mentioned by ≥ 50% of the respondents in the concerning stakeholder group

^d^Importance valued by A) evident risk for implementation, B) potential impact on patients or treatment, and C) relevance for research and development

**Table 2:** Factors mentioned by less than half of the respondents but valued important by A) evident risk for implementation, B) potential impact on patients or treatment, and C) relevance for research and development

**No**	**Implementation factor**	**N**^**a**^	**Categories**	**Value**
**Theme: Patient-related factors**				
1	Shared decision-making methods can be applied to support patients in decision-making	4	Provision of information	B, C
2	Clear information provision of supportive care by means of a patient navigator (*i.e.* specialized nurse) to help patients would be beneficial	5	Provision of information	A, B, C
**Theme: Organizational factors**				
3	FDG-PET/CT to detect nodal status is not performed in all hospitals	5	Identification of patients	A, B
4	The indication for genetic screening is mostly focused on TNBC so there is a risk of missing *BRCA2* mutations (mostly ER + disease and > 40 years)	4	Identification of patients	A, B
5	Data from the Netherlands Cancer Registry shows that not all eligible patients are identified	5	Identification of patients	A, B, C
6	Lack of focus on patients with ER/PR low tumors being referred	2	Referral of patients	A, B
7	The BRCA1-like test is “In-House” developed and thus not CE-marked and patented	5	BRCA1-like test	A, B, C
**Theme: Costs and socioeconomic factors**				
8	Concerns about income continuity and employability of patients	5	Patient costs	B

*ER* + positive Estrogen Receptors, *MLPA* Multiplex Ligation-dependent Probe Amplification, *FDG-PET/CT* Fluorodeoxyglucose Positron Emission Tomography-Computed Tomography, *TNBC* Triple-Negative Breast Cancer

^a^N = Total number of interviews in which this implementation factor has been mentioned

#### Patient-related factors

Clear information provision for patients on the new treatment, a negative sentiment with healthcare professionals due to the history of this treatment in the ‘90 s, and patients sharing their experiences were factors mentioned by more than 50% of one stakeholder group. For example, a patient representative mentioned that:

*“I think the patient information form is not clearly written and has dissuaded some of our patients, it is too long, and too difficult.”*

Additionally, the stakeholders found that it could be beneficial to appoint a patient navigator to improve access to supportive care (*e.g.* physical therapy, psychological therapy) as well as the use of shared decision-making techniques. For example, one healthcare provider said:

*“I am in favor of shared decision-making. However, I do realize that my opinion has an influence on the decision a patient makes. My way of explaining is different than another doctor, I think. And I think that the way you feel about a treatment influences the way you explain the options. In my case, I have preference for the olaparib arm, the PARP inhibitor arm, and might express a more positive view for this arm than the high-dose chemotherapy arm..”*

#### Organizational factors

The most mentioned factors concerning identification and referral were regional multidisciplinary meetings, informing physicians in referring hospitals about this specific subgroup of patients with stage III, HER2 negative, HRD tumors, and the use of an alert in the pathology report to notify healthcare professionals. This alert was automatically generated by the Dutch centralized pathology registration based on the keyword ‘triple-negative breast cancer’. Regarding the organization of HDCT, stakeholders noted that clear communication, well-defined responsibilities, and multidisciplinary collaboration within hospitals, were important factors. As one healthcare provider formulated it:

*“I think collaboration between the hematology department and the medical oncology department could cause implementation issues. In our hospital, the hematology department takes care of the treatment, but if this treatment becomes standard of care in breast cancer, the treatment will probably be moved to the medical oncology department. It is very important to implement the treatment over the different departments carefully. This will only be a small piece of daily care for medical oncologists and we shouldn’t be negligent and implement it haphazard, because that will discourage patients.”*

Moreover, important factors mentioned by some stakeholders were that the identification of all eligible patients may remain a challenge, even after completing the study in case of positive results. The implementation of an FDG-PET/CT during the pre-neoadjuvant therapy work-up, genetic screening of *BRCA2* mutations, and more focus on patients with ER/PR low tumors being referred may systematically improve the identification of patients and ensure equitable access to the treatment in all regions of the Netherlands. For example, one healthcare provider stated:

*“Another thing that is going wrong in the Netherlands is the way patients are staged. In some centers, a PET-CT is always used, while in other centers this is not the case..”*

Furthermore, many stakeholders mentioned that the optimal timing and duration of supportive care is unknown, that patients may benefit from physical therapy, and that seeing the same patient navigator or “buddy” specialized in supportive care would be beneficial. One patient mentioned for example that:

*“I thought it was difficult that I could not continue [talking] with the person I saw at the beginning of the SUBITO trial, when I was admitted in to the hospital. But otherwise, it is fine, the breast care nurses were for example very accessible.”*

Lastly, often-mentioned organizational factors included the centralization of the specialized care (*e.g.* apheresis and stem cell rescue) where necessary and decentralization where possible, to perform the BRCA1-like test in more centers, requirement of experience with the treatment, and the capacity of beds, personnel, and apheresis machines.

#### Clinical factors

Some of the side-effects and adverse events most mentioned by the interviewees were an increase in cardiovascular diseases, effects on cognition, fertility, and psychological problems. Of note, almost all healthcare providers mentioned that high toxicity, intense treatment is acceptable when prognosis significantly improves. As one healthcare provider mentioned:

*“I think both physicians and patients realize that they [the patients] have a poor prognosis, which is why there is a willingness on both sides to go further and opt for a toxic treatment, certainly also because it concerns rather healthy, young women.”*

#### Costs and socio-economic factors

No implementation factors concerning cost were mentioned by at least 50% within one stakeholder group. However, it was mentioned that the scarce availability of thiotepa and the potential commercialization of the BRCA1-like test may increase treatment costs. Moreover, productivity losses, the cost of supportive care, hospital-admitted days, and treatment discontinuation were factors mentioned by the interviewees as potential cost-drivers. Last, some patients and healthcare providers mentioned that some patients may experience concerns about income continuity and employability. For instance, one patient stated that:

*“Well, I am lucky because I am a member of a bread fund, which means that 50 entrepreneurs pay you money if you get sick. Although you only receive that for 2 years and I have used 14 months of it already so if that [the cancer] will return I do have a big problem, yes. As I only have 10 months left.”*

#### Study-related factors

We identified that additional study-related activities for healthcare professionals and randomization might withhold patients from enrolling in the SUBITO study. Moreover, coverage with evidence development programs, and the use of early transparent dialogue among stakeholders are helpful to improve implementation. One policy-maker emphasized for example that:

*“Well, communication and communicating in a timely manner is key. A lot of time can be saved by communicating in the right way. So, timeliness and clear communication and- what I also often see- are plans with an unrealistic timeframe. This is due to the wish to obtain a certain goal. But it has to be realistic, thus sometimes it might be better if it costs more money or takes more time.”*

Moreover, stakeholders mentioned that the possibility of clear communication of prognoses and treatment plans to patients after study results and additional publications on HDCT will help with acceptance among healthcare providers and patients.

## Discussion

Based on our findings (Tables 1 and 2), the importance criteria in relation to all findings, and the current state of the health technology, we identified eight topics in need of further elaboration to guide optimal implementation. In the following section we discuss these topics, relate it with the literature and provide recommendations.

### Identification of patients: Staging of breast cancer patients with FDG-PET/CT-scans

From our interviews, we received indications that staging with FDG-PET/CT scans for breast cancer patients with lymph node positive or tumors larger than 5 cm may not have been performed as standard of care in all hospitals in the Netherlands. In recent history, guidelines for adjuvant systemic treatment made no distinction between stage II or stage III breast cancer, due to a lack of personalized treatments and companion diagnostics. However, developments in the treatment of BRCA1-like and macrometastatic (N +) breast cancer patients indicate that accurate staging is necessary. Omitting FGD PET/CT scans during staging may sometimes lead to understaging [32]. Therefore, the use of these scans for patients with lymph node positive or tumors larger than 5 cm may increase access for patients to novel treatments, and has been added to the Dutch guidelines since 2017. In light of our finding, we emphasize on the routine use of FGD-PET/CT scans for staging in the above-mentioned group in all hospitals, to ensure identification of all eligible patients for HDCT and thus optimal treatment plans.

### Identification of patients: Genetic screening

The selection of patients is currently mostly focused on patients with *BRCA1* mutations and less on patients with *BRCA2* mutations. Emphasizing on the referral criteria, identified mutations and thus patients potentially benefiting from HDCT might increase. Moreover, the timely identification of *BRCA1* and *BRCA2* mutations might be difficult for some centers. Results of the genetic testing must be available before leukapheresis, which is within six weeks after the first chemotherapy course. In some larger hospitals in the Netherlands, patients are treated with the concept of ‘DNA-first’. In the 'DNA-first' innovation project, the treating physician can request DNA testing for hereditary breast cancer without referral to the clinical geneticist, significantly decreasing the time to a result of the genetic test.

### Referral of patients: Referring stage III, HER2-negative, ER/PR low patients

Most patients referred to SUBITO study centers were diagnosed with triple-negative BC and do not have a known germline *BRCA1* or *BRCA2* mutation. The tumors of these patients are tested for a HRD phenotype using the BRCA1-like test. Retrospective analyses have shown that triple-negative or grade 3 ER/PR low (expression below 50%) breast cancer tumor have a higher incidence compared to other subtypes [14, 15]. However, grade 3 breast cancer patients with ER or PR expression below 50% tumors are currently rarely referred for HDCT, which was also mentioned in the interviews. Therefore, it seems that medical oncologists have linked triple-negative breast cancer with BRCA1-like, but not high-grade ER/PR low tumors. Regional meetings, distribution of pocket cards, more attention for this specific subgroup during multidisciplinary consultation, development of treatment guidelines, and reiterating inclusion criteria to physicians is recommended.

### Organization of HDCT: Communication, cooperation and responsibilities of HDCT within hospitals

Hospitals often consist of complex, unique, socio-technical systems, where communication is key to quality of patient care [33]. One of the challenges for the implementation of HDCT is the multidisciplinary approach with medical oncologists, hematologists, surgeons, radiotherapists, nurses, and pharmacists. For example, hematologists are normally not involved in the treatment of breast cancer patients in the current Dutch standard of care. However, for HDCT, the hematology department is responsible for apheresis, the high-dose chemotherapy, and the autologous stem cell rescue. Despite their experience with these procedures, the treatment strategy in solid tumors is different, with extra treatment modalities in surgery and radiotherapy. Therefore, communication and cooperation between different departments is essential for a relatively small population of patients to prevent operational or logistical issues and in the worst-case medical errors. Hence, clear organizational responsibilities, *i.e.* whom is responsible for each part of the multistep treatment and sharing treatment experiences between hospitals will remain important.

### Nationwide organization: Centralized versus decentralized provision of HDCT

Many of the interviewed healthcare professionals were in favor of centralizing HDCT, since extensive experience may favor patient care. Moreover, there is a lengthy learning curve due to the small group of patients eligible for the treatment and centers have to be accredited by the Joint Accreditation Committee of ISCT and EBMT (JACIE). Currently, in the SUBITO study, ten hospitals are enrolling patients for this treatment. However, in some hospitals the enrolment rate is slow (*e.g.* less than five in two years). To facilitate training, limit cost, increase experience and efficiency, and to reduce clinical variability it may be necessary to set a minimum of breast cancer patients treated with HDCT per year [34]. Contrary, it is recommended that other treatment aspects such as induction chemotherapy and follow-up care will be performed in the patients’ local hospital, to reduce travel distances for patients and to optimize the capacity within comprehensive cancer centers.

### Supportive care: Early integration of supportive care

Implementation of supportive care is overlooked in the implementation of novel cancer treatment and demands additional research [35]. Within the SUBITO study all participating patients should have a consultation with a specialized oncology nurse (*i.e.* patient navigator) before start of treatment, and next to the standard clinical follow-up assessments. These nurses assess patients’ needs for supportive cancer care, provide information on supportive cancer care, and guide patients through the supportive cancer care system [36, 37]. Patients’ needs can be assessed using the validated and reliable distress thermometer [38]. Research shows that patient navigators improve equitable access to early supportive care and is likely to result in direct health benefits such as decreased severity of cancer-related psychosocial issues, physical distress, and pain management [36, 37].

Additionally, an inventory of used supportive care of a subset of patients will be performed in the current ongoing study to investigate any potential differences between arms and to enable recommendations should the treatments become clinical practice. The results should be an integrated part of the treatment and be included in the guidelines.

### Provision of information: Information provision and shared decision-making

The provision of information on HDCT and other treatment options before, during and after the treatment should be communicated clearly to all eligible patients. In 2013, guidelines and tools to promote shared decision-making between patients and healthcare providers have been published by the Dutch Healthcare Institute [39]. This report emphasises the use of shared decision-making and especially when there is a lack of evidence or if there are more than one valid treatment strategies. Moreover, current literature argues that shared decision-making should be the norm in most medical practices due to the ethical imperatives such as autonomy, beneficence, and non-maleficence [40]. To judge whether benefits and risks of HDCT compared to another treatment strategy is balanced from a patient’s perspective they have to be well informed. If both treatment arms become insured care, decision aids may be helpful for informed decision-making, in particular due to the complexity and toxicity of the treatment. The decision aids may be in the form of videos, pamphlets, or web-based tools that describe the options available, help patients to understand these options. In order to identify relevant aspects in (shared) decision making and in view of the earlier debate on this intensive treatment, we intend to conduct a discrete choice experiment among patients and professionals.

### The BRCA1-like test: New EU-wide regulation for in vitro diagnostic medical devices

The BRCA1-like test is an in-house developed and used diagnostic test to identify patients with HRD tumors. On May 2022, the European Union’s In Vitro Diagnostic Regulation (IVDR) will come into force, and before that time a conformity assessment of the in vitro diagnostic medical device is necessary to meet the requirements and comply with the more stringent regulation [41]. The IVDR provides an exception for in vitro diagnostics that are developed and applied entirely in-house, as will probably be the case for the BRCA1-like test. Those in-house procedures and products must comply with the quality system of the institution, there must be a well-documented justification for the exceptional position and the in vitro diagnostic must be different or better than any alternatives already available on the market [41, 42]. Therefore, it may be worthwhile to appoint a dedicated team with the responsibility for regulatory compliance with the IVDR as early as possible for centers that would like to use in-house in vitro diagnostics such as the BRCA1-like test when HDCT becomes standard practice [42].

### Limitations

First, this is a qualitative study, which only delivers level VI evidence. Future research should focus in testing specific hypothesis in quantitative form, *i.e*. studies to quantify the use and effects of supportive care for different treatment plans and specific breast cancer patients. Second, we only interviewed healthcare professionals of SUBITO centers; this may not be representative for all hospitals in the Netherlands. Third, all interviewed stakeholders were from the Netherlands and most healthcare professionals worked in a tertiary hospital, which may limit the generalizability of our findings only to the Dutch healthcare system.

### Conclusion

In anticipation of a positive reimbursement decision, we recommend to take into consideration the highlighted implementation factors to expedite and guide high-quality equitable access to HDCT in the Netherlands. Topics that may need attention are 1) the structural use of FDG-PET/CT scans for accurate staging, 2) awareness of referring *BRCA1/2*-mutated patients and the use of ‘DNA-first’ strategies, 3) awareness to refer stage III, HER2-negative, ER/PR low patients for screening, 4) the early integration of suitable supportive care, 5) the centralization of the apheresis, high-dose chemotherapy and stem cell rescue, and the decentralization of the induction chemotherapy and follow-up care, 6) good multidisciplinary collaboration between and within hospitals to guarantee quality of care for this logistically complex intervention, 7) the methods used to provide high quality and understandable information of this novel treatment for both healthcare professionals and patients by means of for instance shared decision-making techniques and decision aids, and 8) compliance with IVDR for the BRCA1-like test.

## Supplementary Information

**Additional file 1: Appendix A. **Aspects studied in CTA as derived from Douma et al. 2007.

**Additional file 2: Appendix B.** Interview guide (Dutch and English).

**Additional file 3: Appendix C. **Identified themes, categories, and subcategories relevant for the implementation of high-dose chemotherapy according to different stakeholders.

## Data Availability

To ensure the anonymity of the respondents, we will not publish the transcripts in an open repository. Additional quotes on specific topics can be attained via reasonable request to the corresponding author.

## Abbreviations

BRCA1: Breast cancer 1 gene
BRCA2: Breast cancer 2 gene
CED: Coverage with evidence development
CTA: Constructive technology assessment
DNA: Deoxyribonucleic acid
FDG-PET/CT: Fluorodeoxyglucose-positron emission tomography/computerised tomography
HDCT: High-dose chemotherapy with autologous stem cell rescue
HER2: Human epidermal growth factor receptor 2
HRD: Homologous recombination deficient
HTA: Health technology assessment
IVDR: In Vitro Diagnostic Regulation
SUBITO: Substantially Improving the Cure Rate of High-risk BRCA1-like Breast Cancer
