# Supplementary material for: High-dose chemotherapy with stem cell rescue to treat stage III homologous deficient breast cancer: factors influencing clinical implementation
Source: BMC Cancer. 2023 Jan 7;23:26. doi: 10.1186/s12885-022-10412-x (PMC9824989; doi:10.1186/s12885-022-10412-x)
Supplement: Supplementary file 1 — Additional file 1: Appendix A. Aspects studied in CTA as derived from Douma et al. 2007. [file 12885_2022_10412_MOESM1_ESM.docx]

| **Appendix A.** Aspects studied in CTA as derived from Douma et al. 2007 | |
| --- | --- |
| **Parameters** | **Aspects** |
| Clinical | Efficacy, safety, effectiveness, outcomes, and the effect on the population |
| Economic | Cost-effectiveness |
| Patient-related | Social and environmental impact, ethics, acceptability, psychological reactions, patient centredness, and other patient-related aspects |
| Organizational | Diffusion, dissemination, organizational implementation, accessibility/equity, skills/routines, education/training, and other organizational aspects |

**Description of aspects:**

**Clinical aspects** include the efficacy, safety, effectiveness and outcomes of the intervention, and its effect on the population. Efficacy of an intervention is determined by the intervention’s ability to stimulate a quantifiable biological response (Galandrin, Oligny-Longpré & Bouvier, 2007). Safety can be measured by the occurrence and severity of adverse events (Patnaik et al., 2016). Effectiveness is often measured by added life years or quality adjusted life years (QALY) (Drummond et al., 2005).

**Patient-related aspects** address the social and environmental impact, ethics, acceptability, psychological reactions, patient centeredness, and other patient-related aspects. Acceptability of a treatment relates to the extent to “which a patient perceives a treatment to be fair, reasonable, appropriate and un-intrusive for a given clinical problem” (Milosevic, Levy, Alcolado & Radomsky, p. 456, 2015). Patient centeredness is described as understanding the patient as a person (Bombeke et al., 2011).

**Economic aspects** consist of the cost-effectiveness of the technology. The definition of cost-effectiveness used in this research is as follows: “the added improvement in health outcomes relative to cost” (Weinstein & Skinner, p.460, 2010).

**Organizational aspects** are diffusion, dissemination, organizational implementation, accessibility/equity, skills/routines, education/training, and other organizational aspects. Diffusion is the spread of the technology throughout the organization (Peres, Muller & Mahajan, 2010). Dissemination is defined as the process of making stakeholders aware of, adopt and routinely use the technology (Hazen, Sankar & Jones-Farmer, 2012). Accessibility refers to temporal access, which is waiting times to enter a treatment, as well as geographical accessibility, which is distance to travel to receive treatment (Kao et al., 2014; Ratcliffe et al., 2009), in which equity is the aspect that considers whether unequal access is also considered unfair (van Wee & Geurs, 2011).

**References:**

Galandrin, S., Oligny-Longpré, G., & Bouvier, M. (2007). The evasive nature of drug efficacy: implications for drug discovery. *Trends in pharmacological sciences*, *28*(8), 423-430. <https://doi.org/10.1016/j.tips.2007.06.005>

Patnaik, A., Rosen, L. S., Tolaney, S. M., Tolcher, A. W., Goldman, J. W., Gandhi, L., ... & Nasir, A. (2016). Efficacy and safety of abemaciclib, an inhibitor of CDK4 and CDK6, for patients with breast cancer, non–small cell lung cancer, and other solid tumors. *Cancer discovery*, *6*(7), 740-753.

Drummond MF, Sculpher MJ, Torrance GW et al. Methods for the economic evaluation of health care programmes. Oxford: Oxford University Press; 2005.

Milosevic, I., Levy, H. C., Alcolado, G. M., & Radomsky, A. S. (2015). The treatment acceptability/adherence scale: moving beyond the assessment of treatment effectiveness. *Cognitive behaviour therapy*, *44*(6), 456-469.

<https://doi.org/10.1080/16506073.2015.1053407>

Bombeke, K., Van Roosbroeck, S., De Winter, B., Debaene, L., Schol, S., Van Hal, G., & Van Royen, P. (2011). Medical students trained in communication skills show a decline in patient-centred attitudes: an observational study comparing two cohorts during clinical clerkships. *Patient Education and Counseling*, *84*(3), 310-318. <https://doi.org/10.1016/j.pec.2011.03.007>

Weinstein, M. C., & Skinner, J. A. (2010). Comparative effectiveness and health care spending—implications for reform. *The New England journal of medicine*, *362*(5), 460-465.

Peres, R., Muller, E., & Mahajan, V. (2010). Innovation diffusion and new product growth models: A critical review and research directions. *International journal of research in marketing*, *27*(2), 91-106. <https://doi.org/10.1016/j.ijresmar.2009.12.012>

Kao, D., Torres, L. R., Guerrero, E. G., Mauldin, R. L., & Bordnick, P. S. (2014). Spatial accessibility of drug treatment facilities and the effects on locus of control, drug use, and service use among heroin-injecting Mexican American men. *International Journal of Drug Policy*, *25*(3), 598-607. <https://doi.org/10.1016/j.drugpo.2013.12.012>

Hazen, B. T., Wu, Y., Sankar, C. S., & Jones-Farmer, L. A. (2012). A proposed framework for educational innovation dissemination. *Journal of Educational Technology Systems*, *40*(3), 301-321. <https://doi.org/10.2190/ET.40.3.f>

Van Wee, B., & Geurs, K. (2011). Discussing equity and social exclusion in accessibility evaluations. *European Journal of Transport and Infrastructure Research (EJTIR)*, *11*(4). <https://doi.org/10.18757/ejtir.2011.11.4.2940>
