# Supplementary material for: High-dose chemotherapy with stem cell rescue to treat stage III homologous deficient breast cancer: factors influencing clinical implementation
Source: BMC Cancer. 2023 Jan 7;23:26. doi: 10.1186/s12885-022-10412-x (PMC9824989; doi:10.1186/s12885-022-10412-x)
Supplement: Supplementary file 2 — Additional file 2: Appendix B. Interview guide (Dutch and English). [file 12885_2022_10412_MOESM2_ESM.docx]

**Appendix B (Dutch)**

**Interviewhandleiding**

Deze interviewhandleiding zal worden gebruikt voor het houden van interviews ten behoeve van een onderzoek naar de implementatie van hoge dosis chemotherapie met autologe stamceltransplantatie (HDC-SCT) voor stadium III borstkanker patiënten met een homologe recombinatie deficiëntie (HRD). Het doel is om door middel van vragen aan zorgprofessionals, patiënten, beleidsmakers en onderzoekers meer inzicht te krijgen in belemmerende en bevorderende factoren voor de implementatie van HDC-SCT in Nederland. Het onderzoek is onderdeel van het voorwaardelijke toelatingstraject genaamd SUBITO en wordt uitgevoerd door onderzoekers van het Antoni van Leeuwenhoek.

Het interview zal worden opgenomen en uitgeschreven. Namen van organisaties en personen worden weggelaten uit de rapportage om anonimiteit te garanderen. Bent u hiermee akkoord? De vragen tijdens het interview zijn semigestructureerd en opgesteld in samenwerking met Dr. Valesca Retel, Prof. Wim van Harten, en Dhr. Vincent de Jong, M.D.

Het kan zijn dat er onderdelen zijn waar u niet bij betrokken bent of geen mening over heeft. Mocht dit zo zijn dan slaan we deze onderdelen over en gaan we naar het volgend onderwerp.

De vragenlijst hebben we opgedeeld in vijf delen en is gebaseerd op een Constructive Technology Assessment (CTA) kader.

**Deel 1: Algemeen**

Demografische vragen:

1. Wat is uw leeftijd?
2. Voor welke organisatie bent u werkzaam?
3. Wat is uw functie binnen die organisatie?
4. Hoe lang werkt u al met de behandeling hoge dosis chemotherapie met autologe stamceltransplantatie (HDC-SCT)?
5. Wat is uw rol bij de behandeling HDC-SCT?
6. Bent u betrokken bij de SUBITO studie? Wat is uw rol binnen deze studie?

Algemene vragen implementatie:

1. Wat zijn denkt u dat de twee belangrijkste belemmerende factoren voor de implementatie van HDC-SCT?
2. Wat zijn denkt u de twee belangrijkste bevorderende factoren voor de implementatie van HDC-SCT?

**Deel 2: Organisatie**

1. **Identificatie, doorverwijzing, en geschiktheid van patiënten**
2. Hoe worden geschikte patiënten geïdentificeerd in Nederland? Wat is belemmerend voor de identificatie? En wat is bevorderend?
3. Wanneer worden patiënten doorverwezen? Wat is belemmerend voor een goede doorverwijzing? En wat is bevorderend?
4. Hoe worden de doorverwezen patiënten getest op geschiktheid voor HDC-SCT? Wat zijn belemmerde factoren voor het uitvoeren van deze test? En wat is bevorderend?
5. Zijn er onderdelen van de identificatie, doorverwijzing, test op geschiktheid veranderd de afgelopen jaren? Wat zijn de redenen hiervoor?
6. Hoeveel stadium III, triple negatieve, HRD patiënten zijn er ongeveer in Nederland per jaar? Worden alle geschikte patiënten behandeld? Waarom niet?
7. Hoe zal de identificatie, doorverwijzing, en geschiktheid na de SUBITO studie verlopen? Wat zullen de grootste problemen zijn? Wat wordt makkelijker?
8. **Hoge dosis chemotherapie met autologe stamceltransplantatie (HDCT)**
9. Kunt u globaal in stappen beschrijven hoe de behandeling werkt en wat hierbij belangrijk is (in uw ziekenhuis)?

Mogelijke vervolgvragen:

- 1. Wat zijn de meest complexe stappen van de behandeling?
  2. Wie zijn hierbij betrokken?
  3. Wie zijn hierbij uitvoerend en wie is verantwoordelijk?
  4. Hoe gaat de samenwerking tussen hematologie en oncologie?

1. Wat zijn belemmerende factoren bij het uitvoeren van de behandeling? Wat zijn bevorderende factoren bij het uitvoeren van de behandeling? Is de uitvoering van de behandeling veranderd na verloop van tijd? Zo ja, waarom?
2. Moesten er professionals getraind worden en/of apparatuur aangeschaft worden voor deze behandeling?
3. Hoe zal de behandeling na SUBITO verlopen? Verwacht je nieuwe belemmerende of bevorderende factoren betreft de organisatie na de studie?
4. Hoe stelt u zich de beste organisatie van de behandeling voor na SUBITO op nationaal niveau?
5. **Ondersteunende zorg**
6. Verandert de revalidatie en/of ondersteunende zorg van patiënten voor, tijdens, en/of na de behandeling HDC-SCT vergeleken met standaard chemotherapie? Wat voor ondersteunende zorg komt er extra bij in vergelijking met standaard dosis chemotherapie? Zal de follow-up langer zijn dan de standaardbehandeling?
7. Hoe wordt deze ondersteunende zorg georganiseerd? Hebben alle (betreffende) ziekenhuizen in Nederland de capaciteit om deze zorg te verlenen?
8. Wat zijn volgens u de grootste belemmerende en bevorderende factoren bij het verlenen van de benodigde ondersteunde zorg voor deze patiënten?
9. **Andere belangrijke onderdelen van HDC-SCT**
10. Wat zijn andere belangrijke organisatorische aspecten van HDC-SCT?

**Deel 3: Klinische en Economische Uitkomsten**

1. **Geschiktheid van patiënten**
2. In hoeverre heeft de BRCA-1 like test invloed op de effectiviteit van de behandeling? Kan dit worden verbeterd (i.e. PPV omhoog)?
3. Verwacht u veranderingen in de kosten voor de identificatie van HRD patiënten (BRCA-1 like test)?
4. **Kosten en effecten van HDC-SCT**
5. Kunt u iets vertellen over de effectiviteit van HDC-SCT in deze populatie? Wat zijn de belangrijkste uitkomstmaten? Hoe is de effectiviteit in vergelijking met de standaardbehandeling? Zal de effectiviteit in de toekomst (nog meer) verbeteren?
6. Hoe verschillen korte- en lange termijn bijwerkingen vergeleken met de standaardzorg?
7. Kunnen de behaalde effecten van HDC-SCT verschillen tussen uitvoerders (in verschillende ziekenhuizen)? Waar zou dit aan kunnen liggen?
8. Wat zijn de grootste kostpenposten van de behandeling? Verwacht u veel veranderingen in kosten als HDC-SCT de standaard zal worden?
9. Wat zijn andere aspecten van de behandeling HDC-SCT die de kosten zullen beïnvloeden?
10. **Ondersteunende zorg**
11. Denkt u dat de kwaliteit van leven voor, tijdens, en/of na de behandeling HDC-SCT wordt verbeterd als er meer ondersteunende zorg wordt gegeven aan deze patiënten? Waarom precies? Welke zorg is hierbij essentieel?
12. Zal dit de effecten van de behandeling HDC-SCT in het algemeen in de toekomst daarom nog (significant) kunnen verbeteren?
13. Verwacht u veel veranderingen in kosten voor ondersteunende zorg als HDC-SCT de standaard zal worden? Wat zullen de grootste kostenposten zijn?
14. **Andere aspecten**
15. Is het huidige onderzoek voldoende om Nederland/ andere landen te overtuigen van de effectiviteit indien de doelen worden behaald?
16. Zijn er andere aspecten die de kosteneffectiviteit (kosten en/of effecten) van de behandeling HDC-SCT in het algemeen beïnvloed?
17. Denkt u dat de kosteneffectiviteit (dus kosten en/of effecten) nog veel verbeterd kan worden of potentieel minder wordt?
18. Tot in hoeverre spelen kosten een rol bij de behandeling?

**Deel 4: Patiënt-Gerelateerde Factoren**

1. **Acceptatie patiënten**
2. Wat zijn de redenen voor patiënten om HDCT te willen ondergaan in plaats van de standaardbehandeling?
3. Wat zijn mogelijke andere redenen die belangrijk kunnen zijn voor patiënten?
4. Wat zijn aspecten voor patiënten om de behandeling niet te willen ondergaan, en voor de standaardbehandeling te kiezen?
5. Wat zouden mogelijke andere redenen kunnen zijn?
6. Hoe wordt er in uw ervaring gereageerd op het nieuws dat een patiënt de behandeling HDC-SCT gaat krijgen? Is dit gesprek een belangrijk onderdeel van de behandeling?
7. Wat zijn andere belangrijke aspecten vanuit het perspectief van de patiënt bij het krijgen van de behandeling?
8. **Acceptatie zorgverleners**
9. Wat denkt u dat de criteria zijn voor zorgprofessionals om wel of niet de behandeling HDC-SCT voor te schrijven? In andere woorden, welke afweging moeten zorgprofessionals nemen voor dit besluit?
10. Voorziet u problemen voor oncologen in de acceptatie en/of adaptatie van de behandeling HDC-SCT? Waarom wel of niet?
11. Wat zijn bevorderende factoren voor de adaptatie en acceptatie van de behandeling HDC-SCT?
12. Wat zijn belemmerende factoren voor de adaptatie en acceptatie van de behandeling HDC-SCT?
13. Zouden er in sommige gevallen de behandeling niet voorgeschreven kunnen worden binnen de doelgroep door oncologen (drop-outs)? Zo ja, bij welke patiënten niet en om welke redenen? Ziet hier nog verbetering in?
14. **Media**

1. Welke rol denkt u dat de media gaat spelen bij de acceptatie van de behandeling bij patiënten?

1. Welke rol denkt u dat de media gaat spelen bij de acceptatie van de behandeling bij zorgprofessionals?
2. **Juridische en ethische factoren**
3. Zijn er juridische problemen rondom deze behandeling? Zou dit belemmerend voor de implementatie van de behandeling kunnen zijn?
4. Speelt ethiek een rol bij de implementatie van de behandeling?
5. Zou de behandeling door ethische overwegingen niet ingevoerd kunnen worden (number needed to harm)?

**Afsluiting:**

Waar zijn we over 5 jaar?

- Noem 2 scenario’s;
- Wat zijn bevorderende en belemmerende factoren bij deze scenario’s?

Heeft u nog aanvullende opmerkingen? Kunt u iets ‘out of the box’ verzinnen belangrijk voor de implementatie van HDC-SCT waar wij nog niet aan gedacht hebben?

Wie raadt u aan om nog meer te interviewen?

Bedankt voor uw deelname! Als u vragen hebt na dit interview dan kunt u contact opnemen met Joost Verbeek ([j.verbeek@nki.nl](mailto:j.verbeek@nki.nl)).

**Appendix B (English)**

**Interview guide**

This interview guide will be used to conduct interviews for a study on the implementation of high-dose chemotherapy with autologous stem cell transplantation (HDC-SCT) for stage III breast cancer patients with homologous recombination deficiency (HRD). The aim of this interview is to gain more insight into factors deemed important for the implementation of HDC-SCT in the Netherlands by means of questions to healthcare professionals, patients, policymakers, and researchers. The research is part of the coverage with evidence development trajectory named SUBITO and is being conducted by researchers from the Antoni van Leeuwenhoek hospital/institute.

The interview will be recorded and transcribed. To guarantee anonymity of the interviewees, names of organizations and persons will be omitted from the report. Do you agree with these terms and conditions? The interview will be semi-structured and is prepared in collaboration with Dr. Valesca Retel, Prof. dr. Wim van Harten, and Mr. Vincent de Jong, M.D.

It is possible that during the interview you will not be able to answer some of the questions due to lack of experience or prior knowledge. In this case, we will skip these parts and move on to the next topic.

There may be questions in which you are not knowledgeable or have no opinion about. If this is the case, these topics will be skipped and we will move on to the next topic.

We have divided the questionnaire into five parts and it is based on a Constructive Technology Assessment (CTA) framework.

**Part 1: General questions**

Demographic questions:

1. What is your age?

2. For which organization do you work?

3. What is your role within that organization?

4. How many years or months of experience do you have with high-dose chemotherapy with autologous stem cell transplant (HDC-SCT)?

5. What is your role in regard to the treatment HDC-SCT?

6. Are you involved in the SUBITO study? What is your role within this study?

General questions implementation:

7. What are in your opinion the two main barriers for the implementation of HDC-SCT?

8. What are in your opinion the two main facilitators for the implementation of HDC-SCT?

**Part 2: Organization**

**A. Patient identification, referral, and eligibility**

1. How are eligible patients identified in the Netherlands? Can you name factors that may be barriers for identification? And factors that facilitate identification?
2. At what point are patients referred? Can you name factors that may be barriers for referral? And factors that facilitate referral?
3. How are the referred patients screened for HDC-SCT eligibility? Can you name factors that may be barriers for this test? And factors that facilitate this test?
4. Were there any changes in identification, referral, or eligibility screening in recent years? If so, what was the reason for these changes?
5. Do you know how many stage-III, triple negative, HRD patients there are in the Netherlands yearly? Do you think that all suitable patients are treated in the SUBITO? If not, why do you think this is?
6. If the inclusion period of the SUBITO study is over, how do you think that identification, referral, and eligibility screening will be done? What aspects do you expect to become easier?

**B. High-dose chemotherapy with autologous stem cell transplantation (HDC-SCT)**

1. Can you describe the different steps required during treatment and what the key elements (in your hospital) are?

Potential follow-up questions:

a. What are the most complex steps of this treatment?

b. Which departments are involved?

c. Who is responsible for these steps and who carries them out?

d. Can you describe the collaboration between haematology and medical oncology?

1. Can you name factors that may be barriers in carrying out the treatment? Can you name factors that may be facilitating in carrying out the treatment? Has the treatment changed over time? If yes, why?
2. Did professionals need to be trained and/or equipment purchased for this treatment?
3. If the inclusion period of the SUBITO study is over, how will the treatment proceed after the end of trial? Do you expect any barriers or facilitators regarding the organization when the study is finished?
4. How would you envision organization of treatment on a national level after completion of the SUBITO study?

**C. Supportive care**

1. Does rehabilitation and/or supportive care of patients before, during, and/or after HDC-SCT treatment differ compared to standard chemotherapy? What kind of supportive care is added compared to standard dose chemotherapy? Will patients have a longer follow-up period than patients treated with standard treatment?

2. How is supportive care organized? Do all (relevant) hospitals in the Netherlands have the capacity to provide this care?

3. What are in your opinion, the greatest barriers and facilitators in providing the required supportive care for these patients?

**D. Other important parts of HDC-SCT**

1. Can you name other important organizational aspects of HDC-SCT?

**Part 3: Clinical and Economic Outcomes**

**A. Costs and effects of patient identification**

1. To what extent does the BRCA-1 like test influence the effectiveness of treatment? Can this be improved?
2. Do you expect changes in costs for the identification of HRD patients (BRCA-1 like test)?

**B. Costs and effects of HDC-SCT**

1. Can you talk about the effectiveness of HDC-SCT in this population? What are the main outcome measures? How is the effectiveness compared to standard of care? Will effectiveness improve (even more) in the future?
2. How do short-term and long-term side effects differ in comparison to standard care?
3. Can the achieved effects of HDC-SCT differ between physicians (in different hospitals)? What could be the cause of this?
4. Can you name the biggest costs during this treatment? Do you expect the costs to change significantly if HDC-SCT becomes the standard of care treatment?
5. Can you name other aspects of HDC-SCT treatment that will influence the costs?

**C. Supportive care**

1. Do you think that quality of life before, during, and/or after HDC-SCT treatment will improve if increased supportive care is given to these patients? If so, why exactly? What care is essential here?
2. Will this –the increased supportive care- (significantly) improve the effects of the HDC-SCT treatment in the future?
3. Do you expect changes in the costs of supportive care if HDC-SCT becomes the standard of care treatment? What will be the largest cost item?

**D. Other aspects**

1. If the study turns out to be positive, will it be sufficient to convince the Netherlands/other countries of the benefit?
2. Can you name other aspects that influence the cost-effectiveness (costs and/or effects) of HDC-SCT treatment in general?
3. Do you think the cost-effectiveness (i.e. costs and/or effects) can fluctuate (i.e. increase or decrease)?
4. To what extent does costs play a role in this treatment?

**Part 4: Patient-Related Factors**

**A. Patient acceptance**

1. Can you name reasons for patients to opt for treatment with HDCT over of the current standard of care treatment?
2. Can you name other reasons that may be important to patients?
3. Can you name reasons for patients to forgo the HDCT treatment and opting for the standard treatment?
4. Can you name more reasons?
5. In your experience, how do patients respond to the news that she/he will receive HDC-SCT treatment? Is this conversation an important part of the treatment?
6. Can you name, from a patient’s point of view, other important aspects concerning the treatment?

**B. Acceptance of healthcare providers**

1. Can you name factors important to healthcare professionals to prescribe or not prescribe HDC-SCT treatment? In other words, what considerations should healthcare professionals take into account?
2. Do you foresee problems for medical oncologists in the acceptance and/or adaptation of HDC-SCT treatment? If not, why; If yes, why?
3. Can you name factors that could facilitate the adaptation and acceptance of the HDC-SCT treatment?
4. What factors are barriers in the adaptation and acceptance of HDC-SCT treatment?
5. Are there circumstances in which the targeted population is not treated with HDC-SCT by medical oncologists (drop-outs)? If so, in which patient groups, and for what reasons? Is there still room for improvement here (to lower drop-outs)?

**C. Media**

1. Do you think the media plays a role in patient acceptance of HDC-SCT treatment?
2. Do you think the media plays a role in the acceptance of HDC-SCT treatment among healthcare professionals?

**D. Legal and ethical Factors**

1. Are there any legal issues regarding this treatment? Could this hinder the implementation of the treatment?
2. Do ethics play a role in the implementation of this treatment?
3. Is it possible that the treatment will not be introduced due to ethical considerations?

**Closing:**

Where will we be in 5 years?

- Can you describe us 2 scenarios?

- What are facilitators and barriers in these scenarios?

Do you have any additional comments? Can you think of something 'out of the box' important for the implementation of HDC-SCT that we have not yet thought of?

Who else do you recommend interviewing?

Thanks for your participation! If you have any questions after this interview, please contact Joost Verbeek (j.verbeek@nki.nl).
