# Supplementary material for: High-dose chemotherapy with stem cell rescue to treat stage III homologous deficient breast cancer: factors influencing clinical implementation
Source: BMC Cancer. 2023 Jan 7;23:26. doi: 10.1186/s12885-022-10412-x (PMC9824989; doi:10.1186/s12885-022-10412-x)
Supplement: Supplementary file 3 — Additional file 3: Appendix C. Identified themes, categories, and subcategories relevant for the implementation of high-dose chemotherapy according to different stakeholders. [file 12885_2022_10412_MOESM3_ESM.docx]

| **Appendix C. Identified themes, categories, and subcategories relevant for the implementation of high-dose chemotherapy according to different stakeholders** | | | | | | | | | |
| --- | --- | --- | --- | --- | --- | --- | --- | --- | --- |
|  | | **Healthcare professionals (n=11)*** | | **Patient(representatives) (n=5)** | | **Policy-makers (n=4)** | | **Researchers (n=3)** | |
| **Theme 1: Patient-related aspects** | |  | |  | |  | |  | |
| **Category 1: Ethical aspects** | | Concerns about equity (*e.g.* financial issues, travel distances, literacy, eligibility criteria) (4/11) | | Concerns about equity (financial issues, travel distances, literacy, eligibility criteria) (1/5) | | Concerns about equity (*e.g.* financial issues, travel distances, literacy, eligibility criteria) (1/4) | |  | |
|  | |  | |  | |  | |  | |
| **Category 2: Provision of information** | | Clear information provision necessary for this complex treatment via leaflets, visual aids and/or websites (8/11) | | Clear information provision necessary for this complex treatment via leaflets, visual aids and/or websites (3/5) | | Clear information provision necessary for this complex treatment via leaflets, visual aids and/or websites (2/4) | | Clear information provision necessary for this complex treatment via leaflets, visual aids and/or websites (1/3) | |
|  | | Shared decision-making methods can be applied to support patients in decision-making (4/11) | |  | |  | |  | |
|  | | Confidence and competence of explaining the treatment (options) of treating medical oncologists is important for patients (2/11) | | Confidence and competence of explaining the treatment (options) of treating medical oncologists is important for patients (1/5) | |  | |  | |
|  | | Clear information provision of supportive care by means of a patient navigator (*i.e.* specialized nurse) to help patients would be beneficial (3/11) | | Clear information provision of supportive care by means of a patient navigator (*i.e.* specialized nurse) to help patients would be beneficial (1/5) | |  | | Clear information provision of supportive care by means of a patient navigator (*i.e.* specialized nurse) to help patients would be beneficial (1/3) | |
|  | |  | |  | |  | |  | |
| **Category 3: Treatment perception** | | A negative sentiment of high-dose chemotherapy due to the history of the treatment (6/11) | | A negative sentiment of high-dose chemotherapy due to the history of the treatment (1/5) | |  | | A negative sentiment of high-dose chemotherapy due to the history of the treatment (3/3) | |
|  | | An initial fear of ‘high-dose chemotherapy’ might exist among patients and/or healthcare providers (complexity and risk) (2/11) | | An initial fear of ‘high-dose chemotherapy’ might exist among patients and/or healthcare providers (complexity and risk) (1/5) | |  | | An initial fear of ‘high-dose chemotherapy’ might exist among patients and/or healthcare providers (complexity and risk) (1/3) | |
|  | | Treatment preferences of treating medical oncologists might influence treatment perception and hamper patient-centred care (2/11) | |  | |  | |  | |
|  | | Clarity in ESMO guidelines on high-dose chemotherapy (3/11) | |  | |  | |  | |
|  | | Sharing of treatment experiences between patients and treating medical oncologists (2/11) | | Sharing of treatment experiences between patients and treating medical oncologists (4/5) | |  | |  | |
|  | |  | |  | |  | |  | |
| **Theme 2. Organization** | |  | |  | |  | |  | |
| **Category 4: Identification of patients** | | FDG-PET/CT to detect nodal status is not performed in all hospitals (4/11) | |  | | FDG-PET/CT to detect nodal status is not performed in all hospitals (1/4) | |  | |
|  | | The indication for genetic screening is mostly focused on TNBC so there is a risk of missing *BRCA2* mutations (mostly ER+ disease and >40 years) (3/11) | | The indication for genetic screening is mostly focused on TNBC so there is a risk of missing *BRCA2* mutations (mostly ER+ disease and >40 years) (1/5) | |  | |  | |
|  | | The use of a pathology alert system (*i.e.* PALGA-alert) or other alerts to create awareness of ongoing trials (7/11) | | The use of a pathology alert system (*i.e.* PALGA-alert) or other alerts to create awareness of ongoing trials (1/5) | |  | | The use of a pathology alert system (*i.e.* PALGA-alert) or other alerts to create awareness of ongoing trials (1/3) | |
|  | | Data from the Netherlands Cancer Registry shows that not all eligible patients are identified (5/11) | |  | |  | |  | |
|  | | Presentations and newsletters on the study population (refreshing knowledge) (4/11) | | Presentations and newsletters on the study population (refreshing knowledge) (1/5) | |  | | Presentations and newsletters on the study population (refreshing knowledge) (1/3) | |
|  | | Multidisciplinary team meetings with (all) regional hospitals to increase inclusion rates (8/11) | |  | | Multidisciplinary team meetings with (all) regional hospitals to increase inclusion rates (1/4) | |  | |
|  | |  | |  | |  | |  | |
| **Category 5: Referral of patients** | | Educate (referring) medical oncologists about the treatment, trial, eligibility criteria and prognoses (3/11) | | Educate (referring) medical oncologists about the treatment, trial, eligibility criteria and prognoses (3/5) | |  | | Educate (referring) medical oncologists about the treatment, trial, eligibility criteria and prognoses (1/3) | |
|  | | Lack of focus on patients with ER/PR low tumors being referred (2/11) | |  | |  | |  | |
|  | | Planning and screening all on one day (perceived as too much info and hassle for the patient) (1/11) | |  | |  | |  | |
|  | | Advantageous when patients can be seen on short notice in a SUBITO centre (1/11) | |  | |  | |  | |
|  | | Trust relationship between referring and treating (SUBITO) hospital personnel (3/11) | |  | |  | |  | |
|  | | Referring hospitals might have a disincentive to refer patients to a SUBITO centre due to (the fear of) losing patients/money (1/11) | | Referring hospitals might have a disincentive to refer patients to a SUBITO centre due to (the fear of) losing patients/money (1/5) | |  | |  | |
|  | |  | |  | |  | |  | |
| **Category 6: BRCA1-like test** | | The BRCA1-like test is “In-House” developed and thus not CE-marked and patented (1/11) | |  | |  | | The BRCA1-like test is “In-House” developed and thus not CE-marked and patented (1/3) | |
|  | | The BRCA1-like test is robust (1/11) | |  | |  | |  | |
|  | | There might be insufficient/unsuitable tumor block to correctly perform the BRCA1-like test (2/11) | |  | |  | | There might be insufficient/unsuitable tumor block to correctly perform the BRCA1-like test (1/3) | |
|  | | Extra standardized biopsy of high-risk breast cancer patients (1/11) | | Extra standardized biopsy of high-risk breast cancer patients (1/4) | |  | |  | |
|  | |  | |  | |  | | Test of FFPE material instead of fresh frozen (1/3) | |
|  | |  | |  | |  | |  | |
| **Category 7: Organization of HDCT** | | Clear communication, responsibilities, and cooperation between and within departments (*i.e.,* medical oncology, haematology, radiology, surgery, nurses, quality managers & hospital pharmacy) (8/11) | |  | | Clear communication, responsibilities, and cooperation between and within departments (*i.e.*, medical oncology, haematology, radiology, surgery, nurses, quality managers & hospital pharmacy) (1/4) | |  | |
|  | | One dedicated professional, and specialized “buddy system” in supportive care (4/11) | | One dedicated professional, and specialized “buddy system” in supportive care (3/5) | |  | |  | |
|  | | Harvesting enough stem cells is important for the treatment course (2/11) | |  | |  | |  | |
|  | |  | |  | |  | |  | |
| **Category 8: Supportive care** | | Multidisciplinary supportive care is perceived as important (*e.g.* oncologic physiotherapy, psychological help, etc.) (2/11) | | Multidisciplinary supportive care is perceived as important (*e.g.* oncologic physiotherapy, psychological help, etc.) (1/5) | |  | |  | |
|  | | The use of “lastmeter” or other questionnaires to bring forward suitable supportive treatment (3/11) | |  | |  | | The use of “lastmeter” or other questionnaires to bring forward suitable supportive treatment (1/3) | |
|  | | Work together with renowned institutes (*e.g.* Ingeborg Douwes Instituut,) to provide supportive care close to home (2/11) | | Work together with renowned institutes (*e.g.* Ingeborg Douwes Instituut,) to provide supportive care close to home (1/5) | |  | |  | |
|  | | Patient tailored program to meet the patients’ needs (4/11) | | Patient tailored program to meet the patients’ needs (2/5) | |  | | Patient tailored program to meet the patients’ needs (1/3) | |
|  | | Use existing knowledge from the haematology department (1/11) | |  | |  | |  | |
|  | | Refer patients back to GP when possible, to prevent hospitalization of the supportive care (2/11) | |  | |  | |  | |
|  | | Optimal timing, necessity and duration of supportive care for this treatment is unknown (6/11) | | Optimal timing, necessity and duration of supportive care for this treatment is unknown (1/5) | |  | | Optimal timing, necessity and duration of supportive care for this treatment is unknown (1/3) | |
|  | | Capacity problems for mental health care (1/11) | |  | |  | |  | |
|  | | Patients would benefit from oncologic physical therapy (5/11) | | Patients would benefit from oncologic physical therapy (3/5) | |  | |  | |
|  | | Some form of trauma treatment might be beneficial for some patients (1/11) | | Some form of trauma treatment might be beneficial for some patients (1/5) | |  | |  | |
|  | |  | |  | |  | | Difficult to predict who will develop for example neurocognitive problems and will benefit most from supportive care (1/3) | |
|  | |  | |  | |  | |  | |
| **Category 9: Nationwide organization (the Netherlands)** | | Refer patients back to referring hospital when possible (*e.g.* for check-up, supportive care, remaining treatment) (5/11) | |  | | Refer patients back to referring hospital when possible (*e.g.* for check-up, supportive care, remaining treatment) (1/4) | |  | |
|  | | Centralize HDCT for quality purposes (*i.e.* use of accreditation, guidelines, & quality managers)  (8/11) | | Centralize HDCT for quality purposes (*i.e.* use of accreditation, guidelines, & quality managers) (1/5) | | Centralize HDCT for quality purposes (*i.e.* use of accreditation, guidelines, & quality managers) (2/4) | | Centralize HDCT for quality purposes (*i.e.* use of accreditation, guidelines, & quality managers) (1/3) | |
|  | | The BRCA1-like test can be performed in all centres if acquainted with MLPA (7/11) | |  | |  | |  | |
|  | |  | |  | |  | |  | |
| **Category 10: Education** | | Training of professionals on high-dose units (nurses, pharmacy staff etc.) (3/11) | |  | | Training of professionals on high-dose units (nurses, pharmacy staff etc.) (1/4) | |  | |
|  | | Experience on ASCT in the treating centre is required (7/11) | |  | |  | | Experience on ASCT in the treating centre is required (1/3) | |
|  | | Personnel should be trained to perform and interpret the BRCA1-like test (3/11) | |  | |  | | Personnel should be trained to perform and interpret the BRCA1-like test (1/3) | |
|  | |  | |  | |  | |  | |
| **Category 11: Capacity** | | Specific capacity for ASCT, e.g. the amount of apheresis equipment, beds, and trained personnel are important (2/11) | |  | | Specific capacity for ASCT, e.g. the amount of apheresis equipment, beds, and trained personnel are important (2/4) | |  | |
|  | | After the SUBITO study the number of patients will likely increase (no randomization – planning needed) (2/11) | |  | |  | |  | |
|  | |  | |  | |  | |  | |
| **Theme 3: Costs and socioeconomic factors** | | | | | | | | | |
| **Category 12: Healthcare and societal costs** | | In the future the availability and price of Thiotepa is uncertain (only one manufacturer) (3/11) | |  | | In the future the availability and price of Thiotepa is uncertain (only one manufacturer) (1/4) | |  | |
|  | | Granulocyte colony-stimulating factor (G-CSF) might be a cost-driver (4/11) | |  | |  | |  | |
|  | | Societal costs due to loss of productivity of patients (3/11) | |  | |  | | Societal costs due to loss of productivity of patients (1/3) | |
|  | | An inventory of used supportive care before, during and after the treatment (1/11) | |  | |  | |  | |
|  | | Commercialization of the MLPA assay might influence the cost of the BRCA1-like test (4/11) | |  | |  | | Commercialization of the MLPA assay might influence the cost of the BRCA1-like test (1/3) | |
|  | | Accurately assessing whether days of hospitalisation can be decreased (lowers costs, and might be patient-friendly) (3/11) | |  | |  | | Accurately assessing whether days of hospitalisation can be decreased (lowers costs, and might be patient-friendly) (1/3) | |
|  | | Treatment discontinuation due to for example side-effects can be prevented by optimized inclusion criteria (5/11) | |  | |  | |  | |
|  | | Patent of olaparib expires (control group) (3/11) | |  | |  | |  | |
|  | |  | |  | |  | |  | |
| **Category 13: Patient costs** | | Awareness of compensation arrangements (*i.e.* transportation) (1/11) | | Awareness of compensation arrangements (*i.e.* transportation) (1/5) | | Awareness of compensation arrangements (*i.e.* transportation) (1/4) | |  | |
|  | | Concerns about income continuity and employability (3/11) | | concerns about income continuity and employability (2/5) | |  | |  | |
|  | | Coverage of supportive care for breast cancer survivors, and the effect of deductibles (2/11) | | Coverage of supportive care for breast cancer survivors, and the effect of deductibles (1/5) | |  | |  | |
|  | | Dentistry due to toxicity (1/11) | |  | | Dentistry due to toxicity (1/4) | |  | |
|  | | Cost of healthy food (1/11) | | Cost of healthy food (1/5) | |  | |  | |
|  | |  | | Sickness benefits for entrepreneurs (1/5) | |  | |  | |
|  | |  | |  | |  | |  | |
| **Theme 4: Clinical aspects** | |  | |  | |  | |  | |
| **Category 14: Side-effects and adverse events** | | Research on differences between side-effects and adverse events between treatment options remain important (1/11) | |  | |  | | Research on differences between side-effects and adverse events between treatment options remain important (1/3) | |
|  | | Effect of HDCT on cardiovascular diseases (*e.g.*, dyslipidemia, arrhythmia, high blood pressure) (3/11) | | Effect of HDCT on cardiovascular diseases (*e.g.*, dyslipidemia, arrhythmia, high blood pressure) (3/5) | | Effect of HDCT on cardiovascular diseases (*e.g.*, dyslipidemia, arrhythmia, high blood pressure) (1/4) | |  | |
|  | | Effect of HDCT on fertility (6/11) | | Effect of HDCT on fertility (1/5) | | Effect of HDCT on fertility (1/4) | |  | |
|  | | Effect of HDCT on cognition (*e.g.* concentration problems, chemobrain, etc.) (8/11) | | Effect of HDCT on cognition (*e.g.* concentration problems, chemobrain, etc.) (5/5) | |  | | Effect of HDCT on cognition (*e.g.* concentration problems, chemobrain, etc.) (1/3) | |
|  | | Effect of HDCT on patient functioning (*e.g.* effect on work, relationships, etc.) (6/11) | | Effect of HDCT on patient functioning (*e.g.* effect on work, relationships, etc.) (4/5) | | Effect of HDCT on patient functioning (*e.g.* effect on work, relationships, etc.) (2/4) | |  | |
|  | | Fatigue due to cancer (5/11) | | Fatigue due to cancer (2/5) | |  | |  | |
|  | | Effect of HDCT on libido and sexual function (1/11) | | Effect of HDCT on libido and sexual function (1/5) | | Effect of HDCT on libido and sexual function (1/4) | |  | |
|  | | Increased sensitivity for infections (1/11) | | Increased sensitivity for infections (2/5) | |  | |  | |
|  | |  | | Clarity on numbers needed to treat, number needed to harm, and toxicity (1/5) | |  | |  | |
|  | | Unexpected adverse events (1/11) | |  | |  | | Unexpected adverse events (1/3) | |
|  | | Severe adverse events due to the treatment must be monitored (*e.g.* death, hospitalization, etc.) (1/11) | |  | |  | |  | |
|  | | Psychological problems due to the treatment (*e.g.* trauma, depression, anxiety etc.) (6/11) | | Psychological problems due to the treatment (*e.g.* trauma, depression, anxiety etc.) (1/5) | | Psychological problems due to the treatment (*e.g.* trauma, depression, anxiety etc.) (1/4) | | Psychological problems due to the treatment (*e.g.* trauma, depression, anxiety etc.) (1/3) | |
|  | |  | |  | |  | |  | |
| **Category 15: Effectivity of the treatment** | | Overall survival is most important for patients (5/11) | | Overall survival is most important for patients (3/5) | | Overall survival is most important for patients (3/4) | | Overall survival is most important for patients (1/3) | |
|  | | Quality of life after the treatment should also be taken into consideration (6/11) | | Quality of life after the treatment should also be taken into consideration (3/5) | | Quality of life after the treatment should also be taken into consideration (2/4) | |  | |
|  | | No regional differences on the effectiveness of the treatment are currently expected (3/11) | |  | |  | | No regional differences on the effectiveness of the treatment are currently expected (1/3) | |
|  | | Supportive care may help regaining quality of life after the treatment (2/11) | | Supportive care may help regaining quality of life after the treatment (1/5) | |  | |  | |
|  | |  | |  | |  | |  | |
| **Category 16: Intensity of treatment** | | HDCT is an intense but short treatment, compared to longer but less intense treatment (1/11) | |  | |  | |  | |
|  | | A high toxicity, intense treatment is acceptable when prognosis significantly improves (7/11) | | A high toxicity, intense treatment is acceptable when prognosis significantly improves (1/5) | |  | | A high toxicity, intense treatment is acceptable when prognosis significantly improves (1/3) | |
|  | | Previously we treated patients with even higher dose, which let to even more toxicity – however, no patient from this trial regrets having HDC (2/11) | |  | |  | | Previously we treated patients with even higher dose, which let to even more toxicity – however, no patient from this trial regrets having HDC (1/3) | |
|  | |  | |  | |  | |  | |
| **Theme 5: Study-related aspects** | |  | |  | |  | |  | |
| **Category 17: SUBITO study** | | No study-related additional activities for healthcare professionals like study forms (3/11) | |  | |  | |  | |
|  | | Randomization might withhold patients from participating with the SUBITO study (6/11) | |  | | Randomization might withhold patients from participating with the SUBITO study (1/4) | |  | |
|  | | Early transparent dialogue and trust between stakeholders remains important (1/11) | |  | | Early transparent dialogue and trust between stakeholders remains important (1/4) | |  | |
|  | | Clear communication of prognoses and treatment plans will be easier after the study – now remain uncertain (5/11) | |  | |  | |  | |
|  | | Coverage with evidence development helped with coverage, communication between stakeholders, and national awareness of the treatment (1/11) | |  | | Coverage with evidence development helped with coverage, communication between stakeholders, and national awareness of the treatment (1/4)_ | |  | |
|  | | Additional publications on high-dose chemotherapy will help with treatment acceptance among healthcare providers (8/11) | | Additional publications on high-dose chemotherapy will help with treatment acceptance among healthcare providers (1/5) | | Additional publications on high-dose chemotherapy will help with treatment acceptance among healthcare providers (3/4) | | Additional publications on high-dose chemotherapy will help with treatment acceptance among healthcare providers (2/3) | |
| *In total we interviewed sixteen healthcare professionals in 11 separate interviews.  BRCA BReast CAncer gene; ESMO European Society for Medical Oncology; FDG-PET/CT Fluorodeoxyglucose-Positron Emission Tomography-Computed Tomography; G-CSF Granulocyte Colony-Stimulating Factor; HDCT High-Dose Chemotherapy with Autologous Stem Cell Transplantation; TNBC Tripe-Negative Breast Cancer; MLPA Multiplex Ligation-dependent Probe Amplification | | | | | | | | | |
